# Pornography Use Among Adults in Britain: A Qualitative Study of Patterns of Use, Motivations, and Stigma Management Strategies

**DOI:** 10.1007/s10508-025-03112-7

**Published:** 2025-04-03

**Authors:** Wendy G. Macdowall, Ruth Lewis, David Reid, Kirstin R. Mitchell, Raquel Bosó Pérez, Karen J. Maxwell, Feona Attwood, Jo Gibbs, Bernie Hogan, Catherine H. Mercer, Pam Sonnenberg, Chris Bonell

**Affiliations:** 1 Department of Public Health, Environments and Society, London School of Hygiene & Tropical Medicine, 15-17 Tavistock Place, London, WC1H 9SH UK; 2 MRC/CSO Social and Public Health Sciences Unit, University of Glasgow, Glasgow, UK; 3 Institute for Global Health, University College London, London, UK; 4 Oxford Internet Institute, University of Oxford, Oxford, UK; 5 Hastings, UK

**Keywords:** Pornography, Adults, Stigma, Stigma management communication

## Abstract

Pornography use is common but stigmatized. In this study, we present a qualitative analysis of the patterns of, and motivations for, using pornography and how the stigma of using pornography manifests in participants’ accounts. We draw on Meisenbach’s (2010) theory of stigma management communication (SMC) to deepen our understanding of how participants managed potential stigma. Data come from 40 semistructured interviews with a diverse sample of adults from across Britain (aged 18–64 years) on the role of digital technologies in their sexual lives. Despite not being a criterion for recruitment, all participants except six women had used pornography and 18 were regular users at time of interview. Pornography was used primarily to facilitate arousal during solo masturbation, and also for mood control, “me time,” or exploration of one’s sexual identity. Some participants also used pornography with a partner to facilitate arousal and experimentation. An understanding of pornography use as stigmatized was evident in participants’ accounts. In terms of SMC theory, “avoiding” strategies were most commonly employed including: hiding pornography use from others; distancing oneself from the stigma of pornography use; and making favorable comparisons between oneself and other users. Other less frequently employed strategies included: “denying” the existence of stigma, reducing the stigma’s offensiveness through “minimization” (emphasizing that one’s pornography use does not harm others), and “transcendence” (highlighting that pornography use can be a means to a valuable end). The stigma surrounding pornography use may act as a barrier to education interventions addressing pornography and seeking help for problematic use.

## Introduction

This paper draws on qualitative research with British adults to explore the patterns of, and motivations for, using pornography, the stigma arising from this usage and how this is managed. The literature on the use of pornography has not to date explored how the associated stigma is managed. We address this question systematically for the first time.

Representative surveys suggest that most men and many women use pornography (Herbenick et al., 2020; Malki et al., 2021; Rissel et al., 2017). Industry data confirm that use is widespread. Pornhub, one of the biggest pornographic video-sharing platforms, claims to receive over 130 million visits per day (Pornhub, 2023). While use of pornography is common, it remains stigmatized. The implications of this stigma for health and well-being are poorly understood.

Quantitative review-level evidence on the impacts of pornography use on attitudes, behaviors, and health is equivocal, and highlights a number of methodological challenges and limitations which renders drawing firm conclusions difficult (Döring, 2009; Dwulit & Rzymski, 2019; Harkness et al., 2015; Litsou et al., 2020; Pathmendra et al., 2023; Raine et al., 2020). Further, until recent years, the empirical study of porn has predominately centered on men and has been dominated by the “negative effects paradigm” which focuses on quantifying the possible harmful effects (McCormack & Wignall, 2017).

There is now a growing body of qualitative research that explores the views and experiences of a wide range of pornography users, examining why, how, and in what contexts people engage with pornography and the perceived consequences of doing so. Such qualitative studies have included couples (Kohut et al., 2017; Shuler et al., 2021), people of diverse sexualities (McCormack & Wignall, 2017; Wignall, 2019) and, increasingly, women (Ashton et al., 2018; Chadwick et al., 2018; Daskalopoulou & Zanette, 2020; Macleod, 2021a, 2021b; Tholander et al., 2022; Tillman & Wells, 2023). While there are divergences and nuances in the accounts of different users of pornography, there are several common themes that are germane to the present study.

The first theme is that people engage with pornography for reasons extending beyond sexual pleasure and curiosity to include emotional distraction/suppression, stress reduction, boredom avoidance and self-exploration (Bőthe et al., 2021; Smith et al., 2015). The second theme is that context is important; individuals’ engagement and experiences with pornography are not static, varying by time and place (Wignall, 2019). The third theme is that people often have a complex relationship with pornography, evoking conflicting feelings, which can include excitement and pleasure alongside shame and guilt (Macleod, 2021a; Tholander et al., 2022; Tillman & Wells, 2023). Understanding how women in particular navigate pornography has been a recent focus of research, as managing mixed emotions may be a particular challenge for those who take pleasure from pornography but battle with engaging with material that may not align with their own values or wider social norms (Macleod, 2021a; Tholander et al., 2022). Macleod (2021b) highlighted that, for the feminists in her sample, stigma infused all aspects of participants’ experiences with pornography, and presented challenges for ethical decision-making about what pornography to watch. While Macleod’s study (2021a, 2021b), focused on feminist users of pornography, she argued that stigma may have significant consequences for the behavior of users of pornography more generally, and called for future research to explore the broader role stigma plays in experiences of using pornography.

As well as affecting users’ behavior, stigma has the potential to discourage open discussion about pornography use. For example, Tholander et al. (2022) found that many young women in their sample did not feel they could talk to those near them about their pornography use, resulting in “private and silent shame” (Tholander et al., 2022, p. 1826). Stigma also acts as a barrier to help-seeking among those for whom pornography use has become problematic. Sniewski and Farvid (2020) interviewed 15 heterosexual men with self-perceived problematic pornography use. They found that the main barriers to disclosure were the guilt and shame that accompanied both pornography use generally and conversations about their problematic pornography use specifically (Sniewski & Farvid, 2020). In terms of broader public health approaches, reducing shame about pornography use has been identified as being central to providing better education about it (Dawson et al., 2020).

Stigma was defined by Goffman in 1963 as an “attribute that is deeply discrediting” and that diminishes the bearer “from a whole and usual person to a tainted, discounted one” (Goffman, 1963, p. 3). Goffman (1963) argued that people’s experience of stigma differs depending on how conspicuous the stigmatized attribute is. He distinguished between the discredited (those for whom the stigmatized attribute is visible) and the discreditable (those for whom the stigmatized attribute can be concealed). Stigma may manifest as discrimination, prejudice, stereotyping or adverse social judgment. It may be experienced (“enacted”) or anticipated (“felt”), and must be managed (Scambler, 2009).

In this paper, we add a novel contribution to the literature on pornography by drawing on stigma management communication (SMC) theory (Meisenbach, 2010) to deepen our understanding of how adults from the general population experience and manage anticipated or actual stigmatization arising from pornography use. In this theory, stigma management strategies fall into six main categories that people may adopt, determined by whether they accept or challenge public understanding of the stigma (on one axis) and whether they accept or challenge that the stigma applies to themselves (on the other axis). The six main categories are: (1) “accepting” (accepting the existence of the stigma and that it applies to them); (2) “avoiding” (accepting that the stigma exists but rejecting that it applies to them, for example by hiding the stigma attribute); (3) “evading responsibility” (accepting the stigma applies but asserting that one should not be held to account for it); (4) “reducing offensiveness” (accepting that the stigma applies but challenging the stigmatization through “minimizing” its harm or “transcending” its harm by arguing it is beneficial); (5) “denying” (rejecting both the stigma and its application by asserting that the attribute should not be stigmatized); and (6) ignoring/displaying (rejecting the stigma and its application by overtly presenting the attribute to challenge the stigma) (Meisenbach, 2010). Each of these six categories involve a number of communication tactics (Table 1). The theory has been used to understand how online sex workers manage stigma associated with their work (Siegel et al., 2022) but has not, to our knowledge, been applied to understanding the management of stigma associated with pornography use.

**Table 1:** Stigma management communication strategies evident in accounts of adults who watch pornography

	Accept that stigma applies to self	Challenge that stigma applies to self
Accepting public understanding of stigma (status quo)	I. Accepting	II. Avoiding Hide pornography use (secrecy) Distance self from stigma Make favorable comparisons with other users of pornography
Challenging public understanding of stigma (change)	III. Evading responsibility IV. Reducing offensiveness Minimization (does not harm others) Transcendence/reframing	V. Denying VI. Ignoring/Displaying

Adapted from Meisenbach (2010)

The questions that this paper examines are: (1) How do adults from the general population describe their patterns of, and motivations for, using pornography? and (2) How do they experience stigma arising from pornography use and what stigma management communication strategies do they use to manage this?

## Method

### Participants

The characteristics of both the full and analytic sample are summarized in Table 2. We conducted 40 semistructured interviews in May and June 2019, with adults aged 18–64 years who were resident in Britain (England *n* = 25, Scotland *n* = 10, and Wales *n* = 5). Half identified as men and half as women. Eighteen described themselves as single, the remaining 22 were married or in another form of established relationship. Thirty defined their sexual orientation as straight or heterosexual, four as bisexual, two as lesbian, and four as gay. The research was undertaken to examine topics for inclusion in the fourth British National Survey of Sexual Attitudes and Lifestyles (Natsal-4). Half the interviews, conducted by London School of Hygiene & Tropical Medicine (LSHTM) researchers, focused primarily on the role of digital technologies in participants’ sexual lives and secondarily on sexual well-being. In the other half, conducted by University of Glasgow researchers, the focus was reversed. Participants were recruited by a research-recruitment agency, using quota-sampling to ensure variation in terms of age, gender, ethnicity, relationship status, sexual identity, and area of residence/area deprivation.

**Table 2:** Participant characteristics

	All (*n* = 40)	Watched pornography at least once per month (*n* = 18)	Had watched pornography previously (*n* = 16)	Never watched pornography* (*n* = 6)
Gender				
Man	20	13	7	–
Woman	20	5	9	6
Age				
18–24	16	10	2	4
25–34	8	5	2	1
35–44	8	3	4	1
45 and over	6	–	6	–
Ethnicity				
White British	32	17	12	3
Asian/British/Pakistani/Indian	4	–	3	1
Black/British/African	3	–	1	2
Mixed ethnicity	1	1	–	–
Relationship status				
Single	18	11	3	4
In a relationship/married	22	7	13	2
Sexual identity				
Heterosexual	30	12	12	6
Bisexual	4	3	1	–
Lesbian	2	–	2	–
Gay	4	3	1	–

^*^Though all had been exposed to pornography in some form

### Measures and Procedure

Six researchers conducted interviews face to face in venues of participants’ choosing, generally their homes or university offices. In all but one interview, only the interviewer and participant were present; one participant requested a friend be in attendance. Prior to seeking informed consent, participants were provided with study information and given the opportunity to ask questions. We ensured participants understood that: they could choose not to answer any questions; they could stop the interview at any point; data would be anonymized and anonymity protected in reporting.

Interviewers used a guide with questions about the participants’ experience of sexual/intimate relationships, use of digital media generally and in relation to sex (including to access sexual images, videos or other sexual content for arousal). Interviewers probed for modes, frequency, timings, feelings associated, and perceived benefits or problems. Interviews were audio-recorded with participants’ consent and transcribed verbatim. After personal identifiers were removed, transcripts were entered into NVivo to facilitate coding and analysis.

### Data Analysis

We conducted thematic analysis of the data, involving both inductive and deductive elements. First, we conducted line-by-line open coding, which generated in vivo codes concerning: patterns of pornography use; motivations; and costs and benefits, which were then augmented and refined iteratively. Second, we used axial coding to identify cross-cutting themes, centering on stigma and its management. In this analytic phase, we identified Meisenbach’s (2010) stigma management communication framework as salient to our data, and a useful theoretical vantage point to aid interpretation. This was prompted by the recurrence and prominence of stigma and its management in the accounts of the participants who had used pornography despite our interview guides not including specific questions on this. Third, we subsequently undertook a more focused, theory-driven round of coding, with all the possible different SMC strategies as defined by Meisenbach (2010) as new codes. Data analysis was led by the first author (WM) and included the whole team at different points during the process. Pseudonyms have been assigned to participants to protect confidentiality.

In terms of reflexivity, there was a shared understanding in the team that watching pornography was a common practice. We recognized pornography as diverse, including amateur and professional, and illegal, exploitative and misogynistic but also ethical pornography. We also recognized a lack of clear-cut lines between what is and is not labeled pornography. We were interested in social meanings of pornography and how these shaped orientations to it, and had a shared belief in the validity of people’s accounts of the positive aspects as well as challenges and harms arising from their use.

## Results

Despite not being a criterion for recruitment, 34 participants had used pornography and the analysis presented below is based on their accounts. In particular, we focus on the eighteen participants who reported regular use of pornography. Of the six who said they had never used pornography, all were heterosexual women and all had been exposed to it in some form, for example via social media. We first provide an overview of participants’ patterns of pornography use and their motivations. We then examine pornography use as a stigmatized practice and, drawing on SMC theory, explore the strategies of stigma management evident in participants’ accounts.

### Patterns and Motivations of Use

When describing their use of images, videos or other content for sexual arousal, all participants referred to this as “pornography” or “porn.” Participants distinguished between actively seeking pornography (commonly described as “watching porn”) and being passively exposed to sexually explicit material, for example on social media or through others’ sharing of pornographic memes or brief video clips via WhatsApp groups.

Of the 34 participants who had used pornography, 18 did so regularly at the time of interview, with frequency ranging from once or twice per month to several times per day. Of these, 13 were male, 11 were single, and 10 were aged 18–24 years. Sixteen participants had used pornography previously but were not regular users at the time of interview. Of these, nine were female, 13 were in a relationship, and they spanned the age range (Table 2).

All those who watched pornography regularly did so via online video-streaming platforms. Pornhub was most commonly mentioned, though other sites were referenced (e.g., xVideos, YouPorn). Both men and women reflected on using platforms that were familiar to them, easy to navigate, and free to access. Participants generally accessed pornography via their mobile phones, though some also referred to using laptops or tablets. These devices were seen as offering easy access, portability and, importantly in terms of this paper’s focus, privacy. Eleven of the 18 regular users only watched pornography on their own. The other seven (five men and two women) said that they had also watched with a partner (though less frequently than on their own) and three of these (all men) said that they had only done so once with a partner.

Sexual arousal and pleasure were the most commonly mentioned motivations for engaging with pornography. When watching pornography alone, this was linked by both men and women to its role as an aid to masturbation. For those who had watched pornography with a partner, its role was described both in terms of arousal and, as one participant Adam put it, “spicing your sex life up a bit.” Adam most frequently watched pornography alone but once every couple of months watched it with his long-term partner:

Erm, very, just, you know, when you’ve been together for twelve years you just want to try different stuff and keep it, keep yourselves, I don’t know, try different stuff, as I said. Just because you can get a bit samey, I suppose, after a while. [Adam, 29 years, man, White British, married, straight]

Other motivations identified in our analysis were mood regulation, “me time,” and curiosity, information and education. It was notable that mood regulation was only talked about by men in our sample, who reflected on its role in de-stressing. This was also linked to its function as an aid to masturbation. For example, Kai talked about getting his “head straight” through reaching orgasm which was facilitated by watching pornography:

It’s just to get my head straight. I’ve always felt that, like, cumming does that for me. [Kai, 18–24 years,^1^ man, White British, single, straight]

“Me time” was similarly linked to masturbation. For example, Adam watched pornography once every couple of weeks and, for him, it was “a bit of me time, really, that I kind of deserve if I’ve had a hard week” [Adam, 29 years, man, White British, married, straight]. This sentiment was echoed in Josh’s account; to him watching porn was “*almost like a treat if you like”* [Josh, 22 years, man, White British, in relationship, gay].

In terms of the motivations of curiosity, information and education, watching pornography was not linked to masturbation. In such instances, the motivation, was not, as Amaya put it, “to get off on it,” but rather to satisfy curiosity:

I think like the older I’m getting, I don’t know if I’m getting more perverse and stuff, like. I’ve watched men having sex with each other and stuff, like. I don’t think that’s been like sexual, it’s been more… intrigued... So, I haven’t watched it to get off on it. [Amaya, 38 years, woman, British Asian, single, straight]

Others reflected on learning new things, or on its role when they were younger in exploring their developing sexuality, for example as Emma describes:

No, it’s just, probably something when I was younger, exploring, maybe more exploring my sexuality in terms of what I liked, and what I didn’t like. And now I’m sort of, I know what I like, I know what I don’t like, so I don’t feel the need. [Emma, 32 years, woman, White British, married, lesbian]

### Pornography Use as a Stigmatized Practice

#### Stereotyping of Pornography Users

Stigmatized stereotypes about users of pornography pervaded most accounts among men and women. These included that pornography was something that mainly men, young people and those not having “real sex” engage in. Other participants, however, talked about how pornography had become less stigmatized and that the stereotypes of those who watched pornography have changed, as illustrated in Peter’s account:

Yeah, I don’t think it’s as taboo as it was. Whereas you always had a vision in the past growing up of a man in a mac reaching up and getting a dirty magazine. Dirty old man. It’s not that. [Peter, 42 years, male, White British, married, straight]

One aspect of pornography that was distinguished by men and women was that of the sharing of pornographic images, memes and clips, for example via WhatsApp groups, which was most commonly, though not exclusively, described by heterosexual men in our sample. Many described this practice as entertainment or “banter,” often referring to it as being funny or jokey as distinct from being sexual or arousing. Amaya suggested that the emergence of pornographic memes and clips had “comicalized” pornography, which she felt was contributing to pornography being “not as serious or taboo. It’s more like tongue in cheek.” [Amaya, 38 years, woman, British Asian, single, heterosexual]. Hence, it appeared that, for entertainment and bonding, the sharing of pornographic memes was less stigmatized. The greater stigma appeared to be attached to using pornography for sexual purposes.

#### Ambivalence

Ambivalence about the use of pornography (either current or past) was evident in many of the accounts of both men and women in terms of the mixed emotions that they referred to when describing their experiences with pornography. On the one hand, participants said that watching porn evoked positive feelings for them. On the other hand, they were often left with their own negative feelings that required management. Some of these negative feelings arose from their own disgust at the images viewed. For example, Lexi described how using pornography made her happy at the time but left her feeling guilty, dirty and ashamed:

Yeah, positive for you at the time, but then I always feel a sense of guilt afterwards. Like, I’ll watch it to, like, make me happy at the time, and then afterwards I feel like dirty, like I feel ashamed. And then I think, God, if I feel like that, what the hell do they feel like after they’re having sex with random people and getting videoed? It’s on the Internet. [Lexi, 24 years, woman, White British, in relationship, straight]

Additionally, participants could struggle with stigma arising from concerns about the views of others. For example, Lexi referred to this in terms of her feelings about watching pornography when she had a partner with whom she could have “real sex.” She hid her viewing from him because she thought, “he would be like, “why are you doing that when we could just have sex?””. Lexi’s account also suggests that some of the shame she felt arose from her sense that she was using an unethical commodity involving an exploited workforce. Despite her considerable ambivalence about using pornography, she continued to do so as it facilitated her being able to achieve sexual pleasure that she suggested was less easily reached with a partner.

### Stigma Management Strategies

#### Avoiding

The most common SMC strategies evident in the accounts of both men and women fell into the “avoiding” category in which participants accepted that the stigma of pornography use existed but denied that it applied to them by using tactics such as keeping their pornography viewing secret and distancing themselves from, or making favorable comparisons with, other users.

***Secrecy.*** As noted above, participants generally said that they watched pornography on their own. They also talked about keeping their pornography viewing secret from others, such as partners and family. John watched pornography several times per week, always alone, which he was emphatic about: “Yeah, definitely something I do on my own.” He hid his pornography viewing from his wife, saying he thought that she would be “disgusted… if she watched it, I really do.”

I feel guilty watching it. I feel like it’s supposedly something I shouldn’t be doing. But also it’s, there’s some sort of desire, sexual desire there to watch it. I’m a bit afraid that she’d, what she would feel about me, watching it. And I think maybe that would affect her in terms of what’s wrong with me, “why is he watching that,” that kind of thing. [John, 37 years, man, White British, married, straight]

In his account, John does not challenge the public view of the stigma but sought to avoid this stigma being applied to him. As well as expressing the mixed emotions his pornography viewing evokes, he talked about it as something he feels he should not be doing and fears that his wife will think that there is something wrong with him if she finds out. Hence, by hiding his pornography viewing, he can, as Goffman (1963) argues, “pass” as normal.

Even in the accounts of those who did not feel ashamed of their pornography use, recognition of the stigma in watching pornography was pervasive. Ben, who lived alone and watched pornography every other day, talked about hiding his viewing out of a desire to protect others near to him and avoid the stigma that might ensue.

I’m on my own, you know, and my computer’s my computer and it’s not like I have to hide anything. I will but I don’t have to but it’s sort of a habit, so it’s a weird thing, how I think still in your mind it’s a... still a little bit of a taboo... I wouldn’t go and tell my mum, you know. Now it’s not like it’s an unnatural thing, I just... It’s just embarrassing to other people. Like I don’t care about telling... Well actually, no, saying that, I don’t think I ever really discussed that sort of thing with girlfriends because... but I wouldn’t have a discussion because I’d find it a bit cringy. [Ben, 29 years, male, White British, single, heterosexual]

Attempts to maintain secrecy went beyond simply not disclosing to others, but included attempts at avoiding detection, for example by using incognito modes on search engines, clearing search histories and/or watching on small screens. For Simon, moving to using incognito mode appeared to have the added benefit of not having to then remove the evidence, though he would rather that there was no trace at all:

When I said I used to use it, the just normal Google, I’d always fully clear my history but now like I use incognito mode. You don’t see it so I don’t need to clear it, but you’d probably just rather it was never there. [Simon, 22 years, man, White British, single, heterosexual]

***Distancing.*** Another avoiding strategy evident in participants’ accounts was “distancing.” Participants commonly positioned their own porn use as “normal” and, in doing so, separated themselves from those whose use of porn could be construed as “abnormal,” highlighting that the stigma of watching porn applied more to “distant others” than themselves. For example, participants talked about accessing pornography via platforms which they felt were reputable and watching pornography that they variously described as “normal,” “vanilla” or “innocent,” distancing themselves from those who accessed pornography that was hardcore, extreme or heavy. For example, Thomas, who watched pornography several times per week, stressed the “normalness” of his pornography tastes by describing it as “casual vanilla”:

General intercourse really… nothing too like, erm, hardcore, if I can say that… I’m not into ropes and chains, or someone being whipped... nothing like that ….. just casual vanilla. [Thomas, 25 years, man, White British, single, gay]

Distancing was also evident in Lexi’s account of why she had only ever watched pornography alone. For her, watching with a partner was something that people with “a crazy sex life” would do:

I don’t think I would ever watch it with my partner. Some people do. If they have like a, you know, where they use like toys and have like a crazy sex life, they might. [Lexi, 24 years, woman, White British, in relationship, heterosexual]

John distanced himself in his interview from content that was not what he wanted to see, expressing deep concern about inadvertently accessing material that crossed the boundary into illegality:

Some of the things I kind of see is not what I want to see. I get worried. I also am very, very concerned with watching anything kind of online. And it’s something that really does concern me in the fact I don’t know what I’m going to stumble upon, and whether somebody might not be, for example, age appropriate. You just don’t know. And the thought of stumbling on something and then watching it or then, you know… It having been accessed by my computer… frightens the life out of me, to the point where it makes me not want to access porn. [John, 37 years, man, White British, married, straight]

Another distancing strategy apparent in the accounts of some of the male participants was resisting the notion that pornography was a significant aspect of their identity. For example, Ben watched pornography on his own every other day. He presented this as “a sort of means to an end.” He went on to explain that “it’s not something I’m investing in, it’s not like I’m going to like, ooh bookmark, you know?” [Ben, 29 years, man, White British, single, heterosexual]. This view was echoed in other accounts, including that of Alfie, who watched pornography several times per week:

Just for however long I am masturbating for usually about 10 minutes… I watch the video and I do what I need to do and then I just… Then after I have finished with that, it’s just not really arousing any more. [Alfie, 19 years, man, White British, single, heterosexual].

Thomas, who watched pornography “a few times a week” also appeared to be distancing himself from the notion that it was a significant aspect of his identity as he described how he “wouldn’t go in there just to see what’s new” [Thomas, 25 years, man, White British, single, gay].

***Making favorable comparisons.*** As with the other “avoiding” SMC strategies of secrecy and distancing, making favorable comparisons is focused on avoiding the stigma being applied to the self rather than challenging its existence. This strategy could encompass those making favorable comparisons with other users of pornography or with one’s own past use. For example, John appeared to manage the stigma associated with pornography use by making favorable comparisons with other people (who can get addicted) and with his own previous use (when he recognized that he did rely on it too much):

I don’t think there is any harm in it. Even though I think it’s something that I know that people can get almost addicted to and things like that. And maybe at points I was in my life where I did rely on porn too much to kind of fulfil sexual desires, or satisfactions. Whereas now, whether it’s just because of sex drive thing, in general has gone down, I’m just not as bothered. [John, 37 years, man, White British, married, straight]

In his account, Simon also appears to be making favorable comparisons with others who can get addicted, and who base their sexual relationships on what they watch:

I think, like, you can see people, like, get almost addicted to it, like, and then I know people, like, people say they base their relationships or the way they have sex through what they see online and things like that and I don’t think, I think there should be something that should be a lot more natural than that. [Simon, 22 years, man, White British, single, heterosexual]

### Denying

Several participants, men and women alike, avoided stigma both by arguing against its existence and by denying that it applied to them. Those who used this tactic expressed the view that “everyone” watches pornography. As one participant put it:

If people say they don’t watch porn, they’re lying [Amaya, 38 years, woman, British Asian, single, straight].

Similarly, Peter who used to watch pornography when he was younger, resisted the stigma associated with both the use of pornography and with masturbation, by arguing for their normality:

Masturbation is something you can’t be embarrassed about, it’s something everybody does and the people that say they don’t, you’re kind of like “well, you’re lying to yourself”. But it’s not a taboo subject, it’s something that everybody probably does at some point in their lives, however frequently they do it that’s up to them. But I think that the fact that you can access it [pornography] and you’ve got the means to do it fairly instantly, I think has probably changed the dynamic of masturbation. [Peter, 42 years, male, White British, married, straight]

However, it was also the case that some of the participants who made efforts to deny there was stigma in using porn by stressing its normality, also employed “avoiding” tactics. For example, in his account Simon [22 years, man, White British, single, heterosexual] stressed that “everyone does it don’t they” but also described the lengths he went to keep his porn use secret and employed distancing tactics (as described above).

#### Reducing Offensiveness

Reducing the stigma’s offensiveness is a SMC strategy employed when individuals accept that the stigma applies to them but seek to change how it is seen by others, through “minimization” (efforts to emphasize that one’s pornography use does not harm others) or “transcendence” (efforts to highlight that pornography use can be a means to a valuable end). (Meisenbach, 2010). These strategies were only evident in the accounts of men in our sample. Alex minimized offensiveness by highlighting that use of pornography was a healthy practice, and that the majority of users are not causing or experiencing harm:

I think it’s quite healthy as well… I mean, there’s nothing wrong in doing it, I don’t think. You know, we were put on this earth like, you know, to have partners and have kids. And I think that’s part of the process of doing that... [Alex, 32 years, man, White British, in relationship, straight]

The strategy of transcendence was only evident in Miles’ account. While many participants expressed concern that people may seek to emulate what they see in pornography, Miles reframed pornography as being a means through which people might channel sexual thoughts that might otherwise cause harm:

If it means that the person can get what they want through a visual aid and they don’t have to do something bad to somebody in the world, I’m all for that. If they want to masturbate to something that nobody else would ever consider, that’s fine. [Miles, 38 years, man, White British, single, gay]

## Discussion

### Summary of Key Findings

We have highlighted the complex relationship the men and women in our study had with pornography, which was characterized by feelings of ambivalence and prominently included a sense of stigma and strategies for managing this.

The motivations we found echoed those reported by others (Bőthe et al., 2021; Smith et al., 2015). Sexual arousal and pleasure were most commonly mentioned, and watching pornography alone was linked to its role in aiding masturbation. Watching pornography with a partner was less common among our participants but for those who did, its function was described in terms of enhancing arousal and facilitating sexual experimentation, as has been reported elsewhere (Kohut et al., 2017). Participants also described their use of pornography and masturbation to seek mood control, “me time,” and exploration and creation of their sexual selves and repertoires. However, it was only the men in our sample who talked about pornography’s role in mood regulation, a finding that chimes with recent US research (Sharkey et al., 2020).

While many participants, both men and women, expressed the view that “everybody” watched pornography and that it has become less taboo, the stigma of watching pornography continued to pervade their accounts. Stigma manifested in the mixed feelings that using pornography evoked. On the one hand, most participants said that watching pornography resulted in positive feelings. On the other hand, they were often left with feelings of shame and guilt. This may have reflected the moral incongruence which can affect users of pornography in which users’ behavior is at odds with their moral beliefs (Grubbs & Perry, 2019). Greater stigma was attached to the use of pornography in individual arousal rather than with the sharing of pornographic memes and clips via WhatsApp groups, which was instead framed as entertainment. This might suggest that at least some of the stigmatization associated with solo use of pornography reflects stigmatization of masturbation.

The literature on the use of pornography has not to date explored how the associated stigma is managed. We address this question systematically for the first time. Various stigma management strategies were evident in our participants’ accounts. Most commonly, these involved efforts at “avoiding” that the stigma applied to them through tactics such as keeping their viewing secret and “distancing” themselves by making favorable comparisons with other users of pornography. That “avoiding” strategies were the most commonly found in the accounts of both men and women is perhaps unsurprising given that this is the tactic arguably most germane in contexts where a stigmatized attribute is not visible, so that individuals are, in Goffman’s (1963) terms, only potentially “discreditable” rather than inevitably “discredited”. Participants described their efforts to ensure their pornography viewing went undetected and expressed fears about others finding out.

Additionally, seemingly in order to deflect that the negative societal view of watching pornography applied to them, many participants positioned their own pornography use as “normal,” and in doing so “distanced” themselves from those whose use they construed as “abnormal.” Some male participants employed another form of “distancing” when they resisted the idea that pornography was a cornerstone of their identity, depicting it merely as a means to an end. Several participants sought to “deny” stigma by arguing against both its existence and that it applied to them, for example by making the case that “everyone” watches pornography. Least common strategies, that were only evident in the accounts of some men in our sample, involved “minimization,” where they accepted that the stigma applied to them but sought to change its status (for example, by highlighting that use of pornography was a healthy practice) or “transcendence” (for example, suggesting that it could help people channel sexual thoughts that might otherwise cause harm to others).

### Study Strengths and Limitations

A strength of our study is that our sample was large, and diverse in terms of sexual orientation, age and relationship status. Our research is useful in describing the diversity in the patterns and motivations for pornography use, which would be unlikely to have been found in a study of a more narrowly defined sample of participants. Our findings suggest both some differences in stigma experiences and management between participants according to gender, but also some areas of similarity according to both gender and sexual orientation. Another strength is that participants were recruited to discuss the role of digital technologies in their personal lives and not pornography use specifically, so we were more able to explore the perspectives of a general group of people with some use of pornography rather than those with a particular engagement or interest in it.

We did not purposely frame the questions in our interview guide in terms of stigma and stigma management communication strategies, which were only used to inform our analysis and not data generation. However, qualitative research should generally aim to be participant-centered rather than researcher-centered in the dialog that occurs in an interview. As such, although we had some broad, open-ended questions about pornography use, the conversations were encouraged to go in the direction that participants had most to say about. The fact that we have such rich data despite not including explicit questions in our guide points to the importance of stigma and its management in participants’ accounts of their pornography use. Hence, we see this as a strength of our research. Another strength is the novelty of our analytic framework, to our knowledge, the first application of Meisenbach’s (2010) theory of SMC to analyze accounts of pornography use.

Our findings may have been limited by social desirability bias about a still-stigmatized practice. However, our questions about pornography came later in the interview when there had been time to build rapport, and feelings of comfort and trust. Participants were asked about their use of digital media in relation to sex. Neither our questions nor our analysis were able systematically to assess how the stigma associated with masturbation as opposed to the stigma associated specifically with solo or non-solo uses of pornography is experienced or managed. The influence of religiosity on pornography use was also not systematically explored in the interviews. However, the interviews were with a range of people and aimed to explore factors that influenced their attitudes to their pornography use. That religiosity did not come up in these discussions may point to the lack of influence of religiosity. However, it is possible that participants did not feel comfortable raising this, or it might have been that our sample did not include sufficient diversity in terms of religiosity.

Our analysis did not identify major thematic differences in the accounts by sexual orientation. It may be that this is because motivations, experiences, and strategies are quite similar or it might be that our sample did not contain sufficient LGBTQ+ participants to fully explore this. Finally, although our sample included men and women, all were cisgender.

### Implications for Policy and Research

Our findings highlight the stigma that pervades the use of pornography for both men and women, which may act as a barrier to efforts to provide education about pornography literacy, and to help-seeking for those whose use of pornography has become problematic. Understanding the ways in which people manage this stigma provides an insight into what this stigma means and how it might be challenged. Awareness of moral incongruence might inform how interventions might support individuals to develop better congruence between their beliefs and behaviors in order to alleviate distress (Grubbs & Perry, 2019).

Pornography is now a required topic for statutory relationships, sex and health education in English secondary schools (Department for Education, 2019). Education about pornography could explore how it is stigmatized, how stigma is managed and how this can form a barrier to communication.

In terms of further qualitative research on the stigma associated with use of pornography and how this is managed, future studies could explore how this differs according to age, gender, sexual orientation and ethnicity. Further quantitative research could explore the extent to which stigmatization is implicated in the mental and sexual health harms associated with use of pornography for example via mediation and moderation analyses.

## Data Availability

We are archiving anonymized transcripts with the UK data service to be available to researchers after completion of the Natsal-4 study.

## Footnotes

1 During the interview Kai confirmed he was in the age range 18–24 years but did not provide his exact age.
